# Detection and genomic characterization of an avian influenza virus A/mute swan/Mangystau/1-S24R-2/2024 (H5N1; clade 2.3.4.4b) strain isolated from the lung of a dead swan in Kazakhstan

**DOI:** 10.1128/mra.00260-24

**Published:** 2024-07-22

**Authors:** Kairat Tabynov, Vitaliy Strochkov, Nurlan Sandybayev, Talgat Karibayev, Maxat Berdikulov, Leila Yelchibayeva, Kuantay Zharmambet, Aidana Kuanyshbek, Zauresh Zhumadilova, Kaissar Tabynov

**Affiliations:** 1 International Center for Vaccinology, Kazakh National Agrarian Research University, Almaty, Kazakhstan; 2 Central Reference Laboratory, M. Aikimbayev National Scientific Center for Especially Dangerous Infections, Almaty, Kazakhstan; 3 Kazakhstan-Japan Innovation Center, Kazakh National Agrarian Research University, Almaty, Kazakhstan; 4 Infectious Disease Diagnostic Laboratory, National Reference Veterinary Center, Astana, Kazakhstan; 5 National Collection of Deposited Strains, Almaty Branch of National Reference Veterinary Center, Almaty, Kazakhstan; Katholieke Universiteit Leuven, Leuven, Belgium

**Keywords:** avian viruses, influenza, HPAI, clade 2.3.4.4b, gene sequencing, Kazakhstan, swan, vaccine strains

## Abstract

The influenza virus strain A/mute swan/Mangystau/1-S24R-2/2024 (H5N1; clade 2.3.4.4b) was isolated in embryonated chicken eggs from the lung of a dead swan found around Lake Karakol (Kazakhstan) during a highly pathogenic avian influenza outbreak in 2024. The aim of this study was to characterize the genetic profile of the isolated strain.

## ANNOUNCEMENT

Highly pathogenic avian influenza (HPAI), caused by influenza A viruses of the genus *Alphainfluenzavirus* of the family *Orthomyxoviridae*, includes the fast-mutating H5N1 strain found in multiple bird species. It is both epizootic and panzootic. In 2020, HPAI A(H5N1) clade 2.3.4.4b viruses emerged and spread mainly through migratory birds to Africa, Asia, and Europe (1).

Between 28 December 2023 and 09 January 2024, an outbreak of HPAI (Outbreak ID: ob_129664) struck mute swans around Lake Karakol near the Caspian Sea coast in Mangistau Oblast, Kazakhstan (latitude: 43.438; longitude: 51.3331). A total of 227 dead swans were detected and tested for avian influenza subtype H5 by the National Reference Veterinary Center using real-time PCR with “AmpliSens Influenza virus A-type-H5, H7, H9-FL” kit (Interlabservice, Russia) (2, 3). For further detailed investigation, 12 samples of organs from four dead swans were sent to the International Center for Vaccinology of the Kazakh National Agrarian Research University (KazNARU). All study procedures were approved by the Commission on Bioethics of KazNARU (dated 15 January 2024).

The samples were subjected to homogenization before the extraction process, in accordance with the methods detailed in our earlier publication (4). Nucleic acids from collected samples were processed using the MagMAX Total Nucleic Acid Isolation Kit (Thermo Fisher Scientific, USA). cDNA synthesis, amplification, and primer removal were done with the SeqPlex RNA Amplification Kit (Sigma, USA) to produce cDNA fragments (150–400 nucleotides). Amplified cDNA products were assessed for quantity and quality using NanoDrop and Qubit. Libraries for downstream analysis were prepared using the Ion Plus Fragment Library Kit (Thermo Fisher Scientific). All procedures were conducted following the manufacturer’s protocols without any modifications. Hemagglutination was detected in 9 out of 12 pathologic organ samples (lungs, trachea, liver, heart) from four swans after the first passage in embryonated chicken eggs using a hemagglutination assay (5).

**Fig 1:**
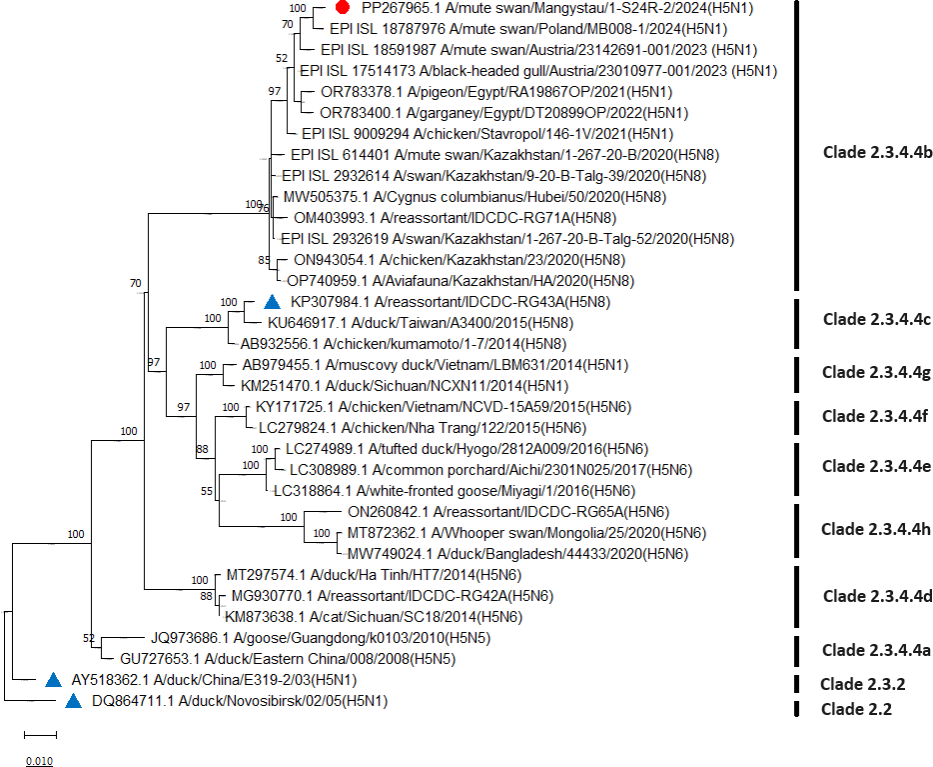
Phylogenetic tree of the HA gene of influenza A(H5Nx) viruses of clade 2.3.4.4 and other viruses circulating the globe, including candidate vaccine viruses (marked with blue triangles). The evolutionary history was inferred by using the Maximum Likelihood method and Kimura 2-parameter model. The tree with the highest log likelihood (−6692.65) is shown. The percentage of trees in which the associated taxa clustered together is shown above the branches. Initial tree(s) for the heuristic search were obtained automatically by applying Neighbor-Join and BioNJ algorithms to a matrix of pairwise distances estimated using the Maximum Composite Likelihood approach and then selecting the topology with superior log likelihood value. This analysis involved 34 nucleotide sequences. Sequences of the HA gene of H5N1 cases in Kazakhstan, WHO-recommended vaccine strains, and other vaccine strains were added to the data set to better understand the phylogenic relationship. There were a total of 1,785 positions in the final data set. Evolutionary analyses were conducted in MEGA11.

Sequencing on the Ion S5 was followed by quality control, trimming, host DNA removal, assembly, and taxonomic classification using EDGE Bioinformatics Pipeline v.2.4.0 (6). This yielded 5,731,152 high-quality reads (average length: 250 bp; range: 50–400 bp) representing the Influenza A virus. We used the A/garganey/Egypt/DT20899OP/2022 (H5N1) strain (GenBank ID: OR793319) as a reference. The assembled genome of A/mute swan/Mangystau/1-S24R-2/2024 (H5N1) is 13,596 base pairs long with a GC content of 44.62%, achieving coding complete sequence coverage exceeding 20,000× depth. BLAST analysis revealed the closest resemblance to HPAI isolated in Egypt in 2022, with 98.59% nucleotide identity in the HA gene to the A/garganey/Egypt/DT20899OP/2022 (H5N1) strain (GenBank ID: OR793319; Table 1). All tools ran with default parameters unless specified otherwise. Evolutionary assessments were conducted using MEGA11 software (7). Fig. 1 shows visual representations of inferred phylogenetic relationships.

**TABLE 1:** Genome characteristics of strain A/mute swan/Mangystau/1-S24R-2/2024 (H5N1)

Gene/ segment	Size (nucleotides)	GC content (%)	Strain with closest relative sequence	Identity at nucleotide level (%)	GenBank accession no.
PB2	2,338	44.40	A/garganey/Egypt/RA20851OP/2022 (H5N1)	97.35	OR783381.1
PB1	2,336	43.92	A/Anas platyrhynchos/Belgium/10402_H195386/2017 (H1N1)	98.24	MT439903.1
PA	2,228	43.76	A/garganey/Egypt/DT20899OP/2022(H5N1)	98.92	OR783399.1
HA	1,769	41.54	A/garganey/Egypt/DT20899OP/2022(H5N1)	98.59	OR783400.1
NP	1,562	47.12	A/garganey/Egypt/DT20899OP/2022 (H5N1)	98.98	OR783401.1
NA	1,456	43.95	A/garganey/Egypt/DT20899OP/2022(H5N1)	98.76	OR783402.1
M	1,022	48.63	A/duck/Egypt/BA20360C/2022(H5N1)	99.02	OP590400.1
NS	885	43.61	A/chicken/Ehime/TU10-2-13/2022 (H5N1)	97.85	LC699188.1

Further genetic analysis at the Kazakhstan-Japan Innovation Center of KazNARU identified the isolated strain A/mute swan/Mangystau/1-S24R-2/2024 as avian influenza virus subtype H5N1, clade 2.3.4.4b, with confirmed HPAI pathotype, based on the PLREKRRKRGLF cleavage site in the HA gene (8).

According to phylogenetic analysis based on the HA gene (Fig. 1), the strain A/mute swan/Mangystau/1-S24R-2/2024 is genetically closest (99%; according to the GISAID database) to strain A/mute_swan/Poland/MB008-1/2024 (H5N1), sampled from a deceased swan (lung sample #1-S24R-2) on 3 January 2024. It is distantly related to several vaccine strains: 90% to A/duck/Novosibirsk/Novosibirsk/02/05 (H5N1; FluProtect vaccine, Russia), 91% to A/duck/China/E319-2/03 (H5N1; Volvac B.E.S.T. AI + ND vaccine, Mexico), and 92% to A/reassortant/IDCDC-RG43A (H5N8; RIBSP’s vaccine, Kazakhstan) used in Eurasian Economic Union countries.

## Data Availability

The complete genome sequence of A/mute swan/Mangystau/1-S24R-2/2024 (H5N1) is available at GenBank under the accession numbers PP267962, PP267963, PP267964, PP267965, PP267966, PP267967, PP267968, and PP267969. The raw sequence reads were deposited under BioProject accession number PRJNA1081595.
